# Over-the-counter Psychosis: A Systematic Review of the Misuse of Antihistamines, Cough Medicines, and Decongestants and the Risk of Developing Psychosis

**DOI:** 10.2174/011570159X344365250114064248

**Published:** 2025-02-18

**Authors:** Alessio Mosca, Stefania Chiappini, Gianluca Mancusi, Andrea Miuli, Carlotta Marrangone, Rita Allegretti, Serena Panichella, Clara Cavallotto, John Martin Corkery, Mauro Pettorruso, Giovanni Martinotti, Fabrizio Schifano

**Affiliations:** 1 Department of Neuroscience, Imaging and Clinical Sciences, “G. D’Annunzio” University, 66100, Chieti, Italy;; 2 Psychopharmacology, Drug Misuse and Novel Psychoactive Substances Research Unit, School of Life and Medical Sciences, University of Hertfordshire, AL10 9EU, Hertfordshire, UK;; 3 UniCamillus International University of Medical Sciences, Via di S. Alessandro 8, Rome, Italy

**Keywords:** Drug abuse, drug misuse, pharming, drug diversion, over-the-counter drug misuse, addiction, OTC, psychosis, schizophrenia, substance-induced-psychosis

## Abstract

**Background:**

The widespread availability and accessibility of over-the-counter (OTC) medicines play a vital role in modern healthcare systems, enabling individuals to manage minor health concerns independently. However, certain OTC medications possess pharmacological properties that render them susceptible to misuse and abuse, including stimulants, laxatives, sedatives, and opiate-containing products. Misuse involves improper dosage, duration, or indication, while abuse entails non-therapeutic use to achieve psychoactive effects or other illicit purposes, potentially leading to dependence and addiction. This review explores the risk of developing psychotic symptoms associated with OTC drug misuse. Synthesizing existing literature, it comprehensively examines the relationship between antihistamines, cough medicines, and decongestants misuse, and the onset of psychotic symptoms.

**Methods:**

A systematic literature review was carried out using Pubmed, Scopus and Web of Science databases through the following search strategy: (“diphenhydramine” OR “promethazine” OR “chlorpheniramine” OR “dimenhydrinate” OR “dextromethorphan” OR “pseudoephedrine” OR codeine- based cough medicines) AND (“abuse” OR “misuse” OR “craving” OR “addiction”) NOT review NOT (animal OR rat OR mouse). For data gathering purposes, the Preferred Reporting Items for Systematic Reviews and Meta-Analyses (PRISMA) was followed. Research methods were registered on PROSPERO (CRD42024527558).

**Results:**

We analysed 46 relevant studies out of an initial pool of 2,677 articles. Key findings indicate that antihistamines, dextromethorphan, and other OTC drugs can induce psychotic symptoms, such as paranoia, hallucinations, and thought disorders when abused. Dextromethorphan is particularly associated with a chronic tendency towards psychosis, whereas other substances more commonly result in acute substance-induced psychosis.

**Conclusion:**

The study underscores the necessity for increased awareness and specific interventions to address the misuse of OTC drugs and their potential to cause significant psychiatric disorders, emphasizing the broader public health implications of such misuse.

## 1 INTRODUCTION

The emergence of postmodern society has led to significant shifts in mental health dynamics, including risk factors, clinical encounters, help-seeking behaviours, and outcomes in psychiatry. Postmodernity's emphasis on present-focused living, influenced by rapid digitalisation and interconnectedness, has altered temporal perceptions, fostering a speed-up society characterized by heightened time pressure and constant change. This acceleration, coupled with these significant social changes, has created an existential condition marked by immediacy and uncertainty, potentially contributing to heightened emotional states and impulsivity among adolescents and young adults [1]. Since the mid-20^th^ century, a unique “drug culture” has formed, rooted in the collective experience of drug use and characterized by its own customs, language, and values. This subculture often reflected and popularized through music and art, provides a sense of identity and belonging, particularly for those who feel alienated from mainstream society. For example, marijuana became associated with jazz musicians in the 1920s, and MDMA with the 1990s rave scene. More recently, a subgroup known as “psychonauts” or “e-psychonauts” has emerged. These individuals engage in the intentional use of psychoactive substances to explore altered states of consciousness and tackle deep spiritual or existential issues, often sharing their experiences and extensive drug knowledge online [2, 3]. Within this context, the phenomenon of ‘pharming’ has gained prominence as a concerning trend, reflecting the intentional misuse of prescription and over-the-counter (OTC) medications for non-medical purposes [4]. The intentional misuse of medications involves using a product other than as prescribed or not in accordance with authorized product information, often driven by perceived rewards such as euphoria or the desire to ‘get high’ [5]. National surveys on drug use and health in the United States (US) have indicated a steady increase in the intentional misuse of prescribed and OTC medications, ranking second only to marijuana use as the nation's most common type of drug use [6]. In Europe, the phenomenon of pharming has been observed through various data sources, including surveys, poison control center records, and pharmacovigilance studies, highlighting the increasing levels of non-medical use and misuse of prescription and OTC medications [7]. The widespread availability and accessibility of OTC medicines constitute a cornerstone of modern healthcare systems, empowering individuals to manage minor health concerns independently. These medications, obtainable without a prescription from registered medical practitioners, are instrumental in promoting self-care and reducing the strain on formal healthcare channels. However, alongside their convenience, OTC medicines harbour potential risks when used improperly, necessitating a nuanced understanding of their misuse, abuse, and dependence. Despite their perceived safety, certain OTC medications possess pharmacological properties that render them susceptible to misuse and abuse. Stimulants, laxatives, sedatives, and opiate-containing products are among the commonly implicated categories, with misuse encompassing improper dosage, duration, or indication. In contrast, abuse involves the non-therapeutic use of these medications to achieve psychoactive effects or other illicit purposes. Complicating matters further, dependence and addiction may ensue, characterized by compulsive use despite adverse consequences and difficulty discontinuing use [8]. The risk of developing psychosis in individuals who abuse OTC drugs is a significant concern within scientific discourse. Despite their widespread availability and perceived safety, certain OTC drugs contain active ingredients capable of influencing the central nervous system and precipitating psychotic symptoms when misused. For instance, antihistamines and agents containing dextromethorphan have been identified as potential culprits, particularly when consumed in high doses or in combination with other substances. This underscores the need for heightened awareness and vigilance regarding the misuse of OTC drugs and their potential psychiatric implications, necessitating further research to elucidate the precise mechanisms underlying this phenomenon and inform targeted interventions [9]. Additionally, psychotic symptoms associated with OTC drug misuse may have several subtypes, depending on the specific substances involved, which may underlie different neurobiological pathways and brain circuits [10, 11]. In light of these considerations, this review investigated the risk of developing psychosis in individuals who abuse OTC drugs. By synthesizing the existing literature, the review comprehensively explores the relationship between OTC medicine misuse, abuse, and the onset of psychotic symptoms. Through examining prevalence trends, identifying implicated medication classes, exploring contributing factors, delineating associated harms, and evaluating intervention strategies, this review provides evidence-based insights into the potential psychiatric implications of OTC drug abuse. By fostering a holistic understanding of the multifaceted nature of OTC medicine misuse, this endeavour strives to inform public health initiatives and promote the well-being of individuals and communities worldwide.

## 2 MATERIALS AND METHODS

### 2.1 Systematic Review Procedures

A systematic electronic search was performed on 2 April 2024 on the following search engines: PubMed, Scopus, and Web of Science (WoS); other relevant papers not resulting from the described search were added from references of included articles. The following search string were used: (“diphenhydramine” OR “promethazine” OR “chlorpheniramine” OR “dimenhydrinate” OR “dextromethorphan” OR “pseudoephedrine” OR codeine- based cough medicines) AND [“abuse” OR “misuse” OR “craving” OR “addiction”] NOT review NOT [animal OR rat OR mouse] NOT “*in vitro*;” in Scopus: [TITLE- ABS-KEY [“Diphenhydramine”] OR TITLE-ABS-KEY [“Promethazine”] OR TITLE-ABS-KEY [“Chlorpheniramine“] OR TITLE-ABS-KEY [“Dimenhydrinate”] OR TITLE- ABS-KEY [“Dextromethorphan”] OR TITLE-ABS-KEY [“Pseudoephedrine”] OR TITLE-ABS-KEY [codeine-based cough medicines] AND TITLE-ABS-KEY [“Abuse”] OR TITLE- ABS-KEY [“Misuse”] OR TITLE-ABS-KEY [“Craving”] OR TITLE-ABS-KEY [“Addiction”] AND NOT TITLE-ABS-KEY [Review] AND NOT TITLE-ABS-KEY [animal] OR TITLE-ABS- KEY [rat] OR TITLE-ABS-KEY [mouse] AND NOT TITLE- ABS-KEY [“*in vitro*”]]; and WoS: [“diphenhydramine” OR “promethazine” OR “chlorpheniramine” OR “dimenhydrinate” OR “dextromethorphan” OR “pseudoephedrine” OR codeine- based cough medicines] AND [“abuse” OR “misuse” OR “craving” OR “addiction”] NOT Review NOT [animal OR rat OR mouse] NOT “*in vitro*.” The systematic review was structured in accordance with the PRISMA [12, 13] guidelines. Identified studies were assessed at title/abstract and full-text levels against eligibility criteria: only original articles written in English that report data on the correlation between OTC misuse and psychosis (delusion, hallucination or schizophrenia); non-original and not written in English that report other substances misuse, or unrelated to psychosis were excluded.

### 2.2 Data Synthesis Strategy

The selection and eligibility phase of the articles were carried out independently by G.M., C.M., R.A., and C.C., followed by a cross-check by A. Mi. and A.M. All discordant cases were evaluated by S.C., M.P., G.M, and F.S. Any unsolved doubts by the team on any of the topics covered in the article were clarified directly from the author, if contactable. The data were collected in a Word table containing the first author’s name and year of publication of the study, study design, demographic variables (gender, age, psychiatric and medical history) details on the drugs taken (dosage and route of administration), detail on psychotic symptomatology [thought disorder, hallucinations, negative symptoms, and outcome), and details of other psychiatric symptoms. The exclusion criteria for both selection phases were: 1) non-original research (*e.g*., review, metanalysis, commentary, editorial, letter to the editor without data available, and book chapter); 2) non-full-text articles (*e.g*., meeting/conference abstract); 3) language other than English; 4) animal/*in vitro* studies; 5) OTC drugs are mentioned only as an example in the context of over-the-counter drugs misuse 6) articles not dealing with the misuse of OTC drugs 7) no psychotic symptoms reported.

### 2.3 Protocol and Registration

Current research methods were approved by PROSPERO (identification code CRD42024527558).

## 3 RESULTS

**Fig. (1):**
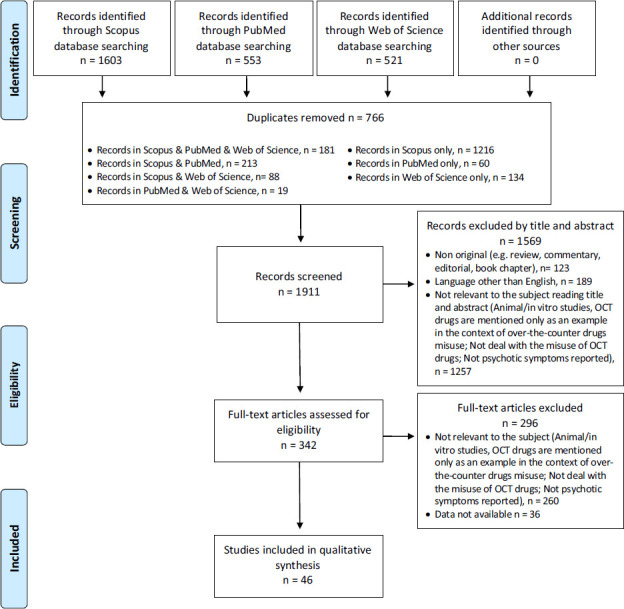
PRISMA flow diagram of the methodology of the systematic literature review.

From a total of 2,677 articles (PubMed = 553; Scopus = 1,603; WoS = 521; other sources = 0), after deduplication (n = 766), 1911 records were screened. Among the articles screened, 1,569 were considered not relevant to the subject after reading the title and abstract, 189 were not written in English, 123 were non-original articles, and 1,257 were excluded because they were animal/*in vitro* studies or OTC drugs were mentioned only as an example within the context of OTC drug misuse, or they did not address the misuse of OTC drugs, or no psychotic symptoms were reported. Of the 342 full-text articles assessed for eligibility, 260 did not match the inclusion criteria for this study, and in 36 instances, data were unavailable. A total of 46 articles were included in the systematic review (Fig. **1**). Findings related to the 46 articles are described in detail and organised by specific molecule and alphabetical order of authors with a focus on psychotic symptomatology (Table **1**). Of the total 46 articles, 27 reported the use of dextromethorphan, 4 of dimenhydrinate, 3 of diphenhydramine, 2 of promethazine, and 10 of other substances (*e.g*., D-norpseudoephedrine, chlorpheniramine, and dextromethorphan triprolidine and pseudoephedrine). Most studies were case reports and case series, except for 3 retrospective studies [14-16] and one survey [17]. Most subjects were male, while the age ranged from a minimum of 14 [18, 19] to a maximum of 69 years old [20]. Regarding psychiatric comorbidity, studies showed a wide variety, with substance use disorder being the most represented diagnosis (N=18), followed by mood disorders (N=12). Regarding medical comorbidities, most studies (N=36) did not report them, except for a few exceptions such as pharyngitis and fibromyalgia [21]; history of seizures, hepatitis C, gastroesophageal reflux disease, hypertension, obesity, and degenerative joint disease [20]; hypertension [22]; myocardial infarction and lymphoma [23]; gastroesophageal reflux disease [24]; migraine [18]. In most studies (N=20), polydrug use was not reported; however, in cases where it occurred, concomitant use of other OTC drugs was reported [14, 16, 22, 23, 25-31]. Secondly, concomitant use of alcohol [15, 29, 31-38] followed by cannabis [15, 19, 26, 31, 34, 38] were reported. Isolated cases of polydrug abuse included opiates [15, 37], cocaine, ecstasy, phencyclidine, ketamine, Lysergic acid diethylamide (LSD) [37], previous methamphetamine abuse [31], a case on methadone maintenance program (150 mg/day) [20]. Regarding affective symptoms, most studies N=7 reported excitatory symptoms (*e.g*., euphoria and sense of well-being) [33, 39-44], and in N=8 cases, manic states [23, 27, 28, 32, 39, 45-47]. Regarding psychotic symptoms, paranoia/paranoid delusion was the most reported thought disorder (N=18), while both visual and auditory hallucinations were found, either alone or in combination (N=32). Negative symptoms of schizophrenia were rarely reported, except in one case with apathy and anhedonia [37]. For further insights into the psychiatric aspect, see Table **1**. In terms of outcome specificity, a second table (Table **2**) is included to delineate the frequency at which OTC drugs led to either acute toxic psychosis induced by the substance or a pronounced exacerbation of a spectrum disorder such as schizophrenia. After excluding studies involving comorbidities with psychotic disorders (schizophrenia spectrum disorders or bipolar disorder), studies were categorized into two groups based on outcome: Substance-induced psychosis and Psychotic onset with a tendency toward chronicity. The table exclusively featured OTC drugs that emerged prominently from the analysis, excluding isolated cases. According to the results presented here, the only substance that led to cases of psychotic onset with a tendency to chronicity was Dextromethorphan, with five cases [21, 32, 42, 45, 48], while sixteen were substance-induced psychosis [20, 22, 23, 25-28, 35-37, 44, 46-51]. As for the other substances, dimenhydrinate, diphenhydramine, and promethazine never resulted in cases of psychotic onset with a tendency to chronicity, but only substance-induced psychosis [18, 19, 29, 52, 53].

## 4 DISCUSSION

Substance-induced psychoses represent a significant subgroup within the spectrum of psychopathological manifestations associated with drug abuse [54]. These psychoses can arise as a result of the assumption of psychoactive substances, including OTC drugs, and are characterized by psychotic symptoms similar to those observed in primary psychiatric disorders such as schizophrenia [9].

Substance-related psychoses pose additional challenges, particularly in distinguishing substance-induced psychopathology from primary psychiatric disorders [1]. In addition to these, intermediate forms of chronic psychoses induced by substances different from schizophrenia itself are also proposed. In this regard, the concept of substance-related exogenous psychosis (SREP) has been proposed to understand persistent forms of substance-induced psychosis, emphasizing the need for a distinct diagnostic framework to address the unique clinical characteristics and treatment responses associated with these conditions [1] - a similar concept is that of “Spiceophrenia” used to refer to those chronic psychoses induced by the use of “Spice”, synthetic cannabinoids [55].

Our findings provide valuable insights into the relationship between OTC drug abuse and the onset of psychosis. The predominant OTC drugs associated with psychotic symptoms include dextromethorphan, dimenhydrinate, diphenhydramine, and promethazine.

Paranoia emerged as the most frequently reported thought disorder among OTC drug abusers. This finding is consistent with the available data on substance-induced psychoses, such as those associated with methamphetamine [56, 57], cocaine [58], cannabis [59], and synthetic cannabinoids. Visual and auditory hallucinations were also commonly observed, either alone or in combination with other psychotic symptoms.

These results are also consistent with observations from episodes induced by other classes of substances [60]. Negative symptoms of schizophrenia, such as apathy and anhedonia, were reported less frequently, indicating a predominance of positive psychotic symptoms in this population.

Affective symptoms associated with OTC drug abuse predominantly include excitatory manifestations such as euphoria and a sense of well-being. Manic states were also reported in some cases. These findings are also in line with what is already known for other substances [61].

In terms of outcomes, dextromethorphan stood out as the only substance associated with cases of psychotic onset with a tendency toward chronicity. In contrast, other substances mainly led to substance-induced psychosis without an evident trend in chronicity. We do not have sufficient information to determine whether chronic psychoses induced by dextromethorphan were actual psychopathological onsets or exogenous SREP. However, this initial distinction underscores the differential impact of specific OTC drugs on the trajectory and persistence of psychotic symptoms, highlighting the need for tailored interventions and treatment strategies based on the substance involved.

Among the studies, a notable gender disparity was observed, with most subjects being male. However, the age range of individuals affected by OTC drug abuse was broad, ranging from adolescents as young as 14 to older adults up to 69 years old. This wide age range emphasizes the importance of considering OTC drug abuse as a potential risk factor for psychosis across different life stages. Even when limiting the analysis to cases of psychotic onset with a tendency toward chronicity, a minimum age of 16 [48] and a maximum of 43 [45]. Polydrug use, although inconsistently reported, emerged as a significant factor in several cases. Concomitant use of other OTC drugs was noted in some cases, indicating a complex pattern of substance abuse among affected individuals. Furthermore, alcohol and cannabis were frequently reported as substances used in conjunction with OTC drugs, indicating potential synergistic effects or patterns of polysubstance abuse.

Healthcare professionals must be vigilant in identifying potential prescription misuse and polydrug abuse, striving to prevent these issues wherever possible. Pharmacists play a key role in reducing drug abuse and should engage in evidence-based practices to detect, understand, and prevent drug diversion and misuse [62]. Additionally, it is crucial to develop policies and practices to address this problem. Educating healthcare providers and the public about the risks of OTC drug misuse, combined with implementing preventive measures, is essential for mitigating the occurrence of drug-induced psychosis and other related health problems [63].

Substance-induced psychoses represent an increasingly concerning phenomenon, demanding a thorough understanding and effective interventions to address the associated challenges. Notably, in cases of substance-induced psychoses, the use of second-and third-generation antipsychotics may be considered as a potential treatment option [64-66]. However, further studies are needed to confirm the efficacy and safety of such therapeutic approaches, aiming to optimize the clinical management of these complex conditions.

## CONCLUSION

In summary, the literature review offers a comprehensive examination of the psychotic manifestations and outcomes linked to over-the-counter (OTC) drug misuse. By detailing patterns of OTC drug misuse, associated comorbidities, symptomatology, and outcomes, this analysis has enhanced our understanding of the complex dynamics between substance misuse and psychosis. This knowledge is crucial for informing clinical practice and guiding future research. OTC drug misuse and abuse not only pose significant risks for inducing psychosis but also represent a major public health concern with profound implications for mental health services and societal well-being. Addressing this challenge necessitates a coordinated effort from clinicians, researchers, and policymakers to reduce the impact of these substances on public health. Enhanced surveillance, focused research, and comprehensive educational outreach are vital to manage the risks associated with OTC drug misuse effectively.

## RECOMMENDATION

Pharmacists and general practitioners play pivotal roles in ensuring patient safety through the sale and prescription of OTC medications. However, a significant limitation in this domain stems from the fact that OTC drug transactions often lack the regulatory oversight typical of prescription medication dispensing. This oversight deficit can lead to inappropriate drug use, increased risk of drug interactions, and potential misuse, particularly among vulnerable populations.

In the UK, OTC medicines are classified into two categories: 'General Sales List (GSL)' items, which can be purchased without pharmacist supervision, and Pharmacy-Only Medicines (POM), which require the presence and supervision of a pharmacist for sale. To ensure patient safety, pharmacists should be vigilant, especially when dispensing POMs, which are often associated with higher risks.

To address these challenges, pharmacy staff and general practitioners should engage in thorough consultations to assess potential abuse patterns in patients. Additionally, they should provide clear information and educational materials on the proper use of OTC medications, potential side effects, and associated risks. Pharmacy staff should also actively monitor abuse patterns and adverse reactions among consumers, reporting any concerns to relevant health authorities to inform broader safety measures.

In international contexts where classifications of OTC medications may differ, it is essential that healthcare professionals follow local regulations while maintaining a consistent emphasis on patient education and safety monitoring. Implementing a system where certain high-risk OTC medications are placed in areas requiring pharmacist interaction prior to purchase can ensure a higher level of oversight and help mitigate misuse.

In terms of 'real-world' clinical practice, research advocates for improving interventions through enhanced surveillance of OTC medication effects, facilitating quicker responses to safety concerns. Policies should also be developed to restrict access to high-risk OTC medications when necessary. For instance, countries should consider adopting flexible drug scheduling laws that can rapidly adapt to the misuse of certain medications. Healthcare professionals and emergency responders require targeted training to identify and manage incidents related to over-the-counter drug misuse effectively.

Public health strategies should focus on harm reduction, employing initiatives such as public education campaigns and evidence-based interventions. International collaboration in this area can help standardize practices and enhance global safety measures.

## Figures and Tables

**Table 1:** Overview of literature cases of over-the-counter drugs misuse: summary of the results related to psychotic symptoms.

**Substance**													
**References**	**Dosage and ROA**	**Study Design**	**Population ** **(N tot)**	**Mean Age (Years)**	**Psychiatric Comorbidity**	**Medical Comorbidity**	**Poly-abuse (Substance)**	**Affective Symptoms**	**Thought Disorder**	**Hallucinations**	**Negative Symptoms**	**Other ** **Psychiatricr Symptoms**	**Outcome**
**Dextromethorphan (DXM)**													
Akerman *et al*., (2010)	Increased use up to 1,440 to 3,840 mg per day, oral	Case report	N = 1 (M)	Age = 17 yrs	ADHD, SUD (alcohol and cannabis)	None	None	Well-being, calmness, weightlessness, increased concentration and focus	NA	Auditory and visual hallucinations	No	A feeling of displacement, cloudy thoughts, and physical stiffness	Resolution of psychotic crisis. Began taking atomoxetine for ADHD
Alam *et al*., (2013)	600 mg daily, oral	Case report	N = 1 (M)	Age = 22 yrs	Cannabis-induced psychotic episode; Schizoaffective disorder (on treatment with antipsychotic)	NA	None	Euphoria	Paranoia; disorganized and intrusive behaviour, poor self-care, fatuous incongruous affect, and irritability, with no clear response to antipsychotic medication	Visual, and auditory hallucinations	No	Dissociation	After psychotic symptoms resolved, maintained remission until took DXM again
Amaladoss and Brien, (2011)	DXM-containing cough syrup administered above the required and recommended dosage, oral	Case report	N = 1 (F)	Age = 20 yrs	Adjustment disorder, insomnia	Pharyngitis and fibromyalgia on treatment with oxycodone, acetaminophen, and clindamycin	None	Mood lability	Bizarre paranoid and somatic delusions, disorganized and pressured speech	Visual and auditory hallucinations	No	None	Partial resolution with olanzapine
Bernstein *et al*., (2019)	1,400 mg over the course of 3 days, oral	Case report	N = 1 (F)	Age = 37 yrs	Long-term misuse of DXM (5 yrs)	NA	None	Manic state with insomnia, euphoria	Grandiose and religious delusions	Auditory hallucinations influencing her behaviour (she used scissors attempting auto-enucleation)	No	None	Resolution of psychotic crisis. Depression treated with citalopram
Butwicka *et al*., (2013)	DXM and pseudoephedrine; Unspecified dose, oral	Case report	N = 1 (M)	Age = 16 yrs	History of misuse of DXM and pseudoephedrine	None	None	NA	Unspecified psychotic symptoms	Unspecified psychotic symptoms	NA	NA	Diagnosed with schizophreniform disorder
Craig, (1992)	Vicks formula 44-D 800 mg/d: 2,400 mg, oral	Case report	N = 1 (F)	Age = 25 yrs	History of OTC drug abuse	None	Phenylpropanolamine and guaifenesin	Labile affect	Tangential speech paranoid psychosis with delusion	NA	NA	Impaired attention and concentration	Discharged uneventfully after receiving supportive care
Dilich and Girgis, (2017)	Many empty bottles of Robitussin^®^ found lying around the house; estimated he was drinking several bottles/day	Case report	N = 1 (M)	Age = 69 yrs	History of SUD (alcohol, opioid, and cocaine) in remission	History of seizures, hepatitis C, gastroesophageal reflux disease, hypertension, obesity, and degenerative joint disease	On methadone maintenance program (150 mg/day)	Mood liability	Paranoid delusions; disorganized thinking with racing thoughts and tangential thought process	Auditory hallucinations	NA	Intoxication delirium; psychomotor agitation; insomnia; unusual and irregular behaviours	Brought to the ED by law enforcement for psychiatric evaluation after family called the police. Became aggressive at staff and was medicated with haloperidol 5 mg and lorazepam 2 mg and subsequently placed in four-point soft restraints. Discharged after three days
Forrester, (2011)	Coricidin^®^ (53.6%); Vicks^®^ and other Proctor & Gamble products (13.0%); Robitussin^®^ (11.4%); and Delsym^®^ (5.2%), oral	Retrospective study	N = 3,421 (M: 60.4%)	Mean age = 13-19 yrs, (69.0%)	NA	NA	DXM+ chlorpheniramine (52.2%); DXM alone (11.5%); DXM+ acetaminophen+ doxylamine+ pseudoephedrine (10.2%); DXM+ guaifenesin (9.4%)	Agitation/irritability (10.1%)	Unspecified psychotic symptoms (5.7%)	Unspecified psychotic symptoms (5.7%)	NA	Agitation/ irritability (10.1%); confusion (8.5%)	A potentially serious outcome was assigned in 46.2% of the cases and 83.1% were managed at a health care facility; death (0.1%)
Ghosh, (2011)	Case 1: 600 mg daily, oral; Case 2: cough mixtures use started 2 yrs before for sinusitis and cough, and then increased the amount to 1 bottle daily (890 mg of DXM); Case 3: DXM, codeine and DPH containing cough mixtures (up to 2 bottles = 1,780 mg of DXM)/month	Case series	N = 3 (2M, 1 F)	Mean age = 21.3 yrs	Case 1: NA; Case 2: tobacco smoker (40 cigarettes daily); Case 3: tobacco smoker (6 cigarettes per day), occasional past use of sleeping pills and hard drugs	Case 1, 2: NA	None	NA	Case 1: paranoid delusions; Case 2: unspecified delusions drowsiness, confusion	Case 1: visual and auditory hallucinations; Case 2: auditory hallucinations	NA	Case 2: Drowsiness, confusion	Clinical stabilization with apparent restoration to baseline
Hapangama and Kuruppuarachchi, (2011)	>120 mg daily, oral	Case series	N = 5 (M)	Mean age = 20.4 yrs	NA	NA	NA	Elevated mood; irritability	Paranoid, and grandiose “delusion	Auditory and visual hallucinations	NA	Social withdrawal; dissociation and depersonalization	Treated with antipsychotics and behaviour therapy programs
Hinsberger *et al*., (1994)	1500 mg/die; oral	Case report	N = 1 (M)	Age = 39 yrs	SUD (alcohol)	NA	Alcohol	Mood fluctuations	Unspecified delusion	Visual hallucination	NA	Aggressive and disruptive behaviour; poor attention span; intense suicidal ideation; restlessness; insomnia	Hospitalization in acute maniac state. Progressive cognitive deterioration and worsening of psychiatric symptoms due to long-term drug abuse
Iaboni and Aronowitz, (1995)	240-720 mg, oral	Case report	N = 1 (M)	Age = 37 yrs	Chronic paranoid schizophrenia; SUD (alcohol)	None	Alcohol	Pleasure	Thought broadcasting	NA	NA	Heightened awareness to environmental stimuli; altered time perception; panic; agoraphobia	Clinical stabilization with apparent restoration to baseline
Jamison and Vasudeva, (2009)	120 mg per day, oral	Case report	N = 1 (F)	Age = 60 yrs	NA	Hypertension	Propoxyphene, hydrocodone	NA	Substance-induced delirium; religious delusions	Visual and olfactory hallucinations	NA	Psychomotor agitation with aggressiveness	Treated with lorazepam, olanzapine, and aripiprazole; discharged in stable condition
Logan *et al*., (2012)	Case 1: intensive DXM use over a period of 12 months (doses of 600-900 mg daily); Case 2: ingested 16 tablets of Coricidin^®^ (430 mg of DXM); Case 3: 22 Coricidin^®^ pills (660 mg); Case 4: 164 pills of Corcidin^®^ (4,920 mg of DXM); Case 5: 300-500 mg of DXM daily	Case series	N = 5 (5 M)	Mean age: 21yrs	Case 1-2: NA; Case 3: SUD (DXM+ chlorpheniramine, LSD, and cannabis); Case 4: NA; Case 5: History of SUD (opioids)	Case 1,2, 3,4, 5: NA	Case 1: None; Case 2: chlorpheniramine, cannabis; Case 3: Cannabis; Case 4: Chlorpheniramine; Case 5: NA	NA	Case 1: DXM-induced paranoia Case 2: attempted suicide due to unspecified psychotic symptoms, attempting to exit the window of the fifth-floor room. Was restrained by friends, but soon after one of them noticed he was not breathing and called paramedics, who pronounced him dead; Case 3: found dead at home (hanging by the neck from the closet doorknob); Case 4: found dead at home; Case 5: DXM-induced psychosis	Case 1: NA Case 2: NA Case 3: NA Case 4: NA Case 5: NA	NA	Case 1: Altered behaviour with auto/ hetero aggressiveness; Case 2: NA; Case 3: NA Case 4: found dead at home; Case 5: abnormal behaviour and aggressiveness	Case 1: He wounded himself to death; Case 2: drug-related death; Case 3, 4: suicide; Case 5: after 2 days of symptomatic treatment, his mental state cleared and his behaviour returned to baseline
Marsh *et al*., (1997)	>237 mg, oral	Case report	N = 1 (F)	Age = 14 yrs	Bulimia; depression; SUD (alcohol, cannabis, cocaine, and LSD)	None	Alcohol, cannabis	NA	NA	Visual hallucinations	Poor concentration, blackouts, abuse	-	Hospitalized for 30 days for intensive psychiatric treatment and started on Prozac 20 mg, and individual/group-therapy for the treatment of her eating disorder and substance abuse
Martinak *et al*., (2017)	Coricidin™ Cough and Cold (1,080-4,000 mg/day total)	Case report	N = 1 (F)	Age = 40 yrs	SUD (alcohol, cannabis, LSD, opioid, benzodiazepine, amphetamines); previous psychotic episode, PTSD, insomnia, depression	NA	Alcohol	Mood lability	Paranoid delusion; suspiciousness	NA	NA	Irritability; aggressiveness	Symptoms remitted following treatment with an antipsychotic and mood stabilizer (olanzapine and valproate). Diagnosis of DXM-induced psychotic disorder
Miller, (2005)	Started from 240 mg daily of DXM syrup, then preferring DXM-containing Coricidin^®^ pills (easier to carry on, to steal from stores and to titrate), max dose 480 mg daily, oral	Case report	N = 1 (M)	Age = 18 yrs	Dysthymia	None	None	“Floaty” feeling, sense of euphoria	NA	Altered perception of time	NA	Mild dissociation, and dysphoria, restlessness and craving after abruptly stopping use	Probably resolution. Naltrexone was used as an anti-relapse agent for DXM dependence
Modi *et al*., (2013)	Four bottles of cough syrup (10 mg each)	Case report	N = 1 (F)	Age = 46 yrs	Mood disorder and SUD (methamphetamine, oxycodone)	NA	Guaifenesin; patient’s urine drug screen, and alcohol level were negative	NA	Paranoia	Auditory hallucination	NA	Abnormal behaviour with auto/ hetero aggressiveness, insomnia	Resolution: diagnosis of Substance-induced psychosis
Polles and Griffith, (1996)	Unspecified dose of Delsym Cough Formula^®^, oral	Case report	N = 1 (M)	Age = 43 yrs	Depression, SUD (opioid) in remission	NA	None	Manic state with euphoria, endless energy, excessive expenses	Paranoid delirium; tachypsychism	Auditory hallucinations	NA	Bizarre behaviour; insomnia	Partial resolution with IM haloperidol. Manic symptoms and psychotic thought steadily diminished after DXM discontinuation
Ritter *et al*., (2019)	DMX; oral	Retrospective, cohort analysis	N = 203 patients were diagnosed with DXM toxicity (F: 112, 55.2%)	Mean age 28.0 ± 20.7 yrs (age range 1-97) (SD=NA)	N = 95 (46.8%) SUD	NA	Alcohol, opiates, and cannabis	Anxiety (18.2%)	Unspecified delusion	Unspecified hallucinations (11.8%)	NA	NA	Prognosis depended on patient age, comorbidity, and polysubstance abuse. There were no fatalities.
Roberge *et al*., (1999)	33.75 mg, oral	Case report	N = 1 (M)	Age = 22 yrs	NA	NA	Pseudoephedrine	Hyper irritability, drug-induced agitated psychosis, and ataxia	Unspecified psychosis	NA	NA	NA	Discharged uneventfully after supportive care
Roy *et al*., (2013)	800 mg per day, oral	Case report	N = 1 (F)	Age = 45 yrs	Opioid dependence, major depressive disorder, and obsessive-compulsive disorder	NA	None	Euphoria. Depression after long-term	Unspecified multiple psychotic episodes	Unspecified multiple psychotic episodes	NA	Long-term use was associated with intense craving, tolerance, and withdrawal symptoms, including severe fatigue, depression, and restlessness	NA outcome. Treatment with topiramate resulted in complete resolution of craving
Sharma *et al*., (2005)	240 mg, oral	Case report	N = 1 (M)	Age = 54 yrs	None	Myocardial infarction and lymphoma	Chlorpheniramine	NA	Acute psychosis with paranoid thoughts	Auditory hallucinations	NA	NA	Resolution of symptoms with haloperidol 5 mg IM; symptoms resolved over 2 days
Stanciu and Penders, (2015)	600 mg, oral	Case report	N = 1 (M)	Age = 20 yrs	NA	NA	Chlorpheniramine	Substance-induced manic toxidrome	Unspecified delusions with pressured speech and grandiosity	Auditory hallucinations,	NA	Agitation	Resolution of symptoms with Risperidone and lorazepam
Walker and Yatham, (1993)	Benylin DM 400 ml daily (600 mg), oral	Case report	N = 1 (M)	Age = 40 yrs	None	None	None	Mania	Present not specified	Present not specified	NA	NA	Resolution of symptoms with small doses of haloperidol
Wolfe and Caravati, (1995)	12-ounce bottles of cough syrup (Robitussin DM^®^), 2,160 mg, oral	Case report	N = 1 (M)	Age = 23 yrs	SUD (alcohol)	None	Alcohol	NA	NA	Unspecified hallucinations	NA	Psychomotor agitation; confused speech	Symptomatic resolution with 50 g activated charcoal orally and IV thiamine and naloxone
Ziaee *et al*., (2005)	75-2,700 mg, oral	Case series	N = 53 (M:.48, F: 5) volunteers who had consumed DXM	Mean age 23.4 yrs, (SD = 1.83).	Depression, anxiety, occasional drug abusers	NA	Alcohol (96.2%), cannabis (88.7%), sedatives (71.7%), LSD (67.9%), morphine 54.7%, ecstasy (52.8%), cocaine (30.2%), heroin (24.5%), phencyclidine (20.7%), ketamine (20.7%), others (24.5%)	Euphoria, dysmnesia, panic disorder, hyperactivity	NA	Auditory and visual hallucinations	Apathy; Anhedonia	Trance, laughing, tongue biting, insomnia, nightmares, hypervigilance, attention deficit, learning impairment, flashback	Symptomatic resolution with restututio ad integrum. No medical support needed
**Dimenhydrinate (DH)**													
Brown and Sigmundson, (1969)	Unspecified dosage, oral	Case report	N = 1 (M)	Age = 18 yrs	None	None	None	Anxiety,	Drug-induced delirium with paranoia, thought incoherence	Visual and auditory hallucinations	NA	Emotional liability, agitation	NA outcome. Treated with IV chlordiazepoxide and benztropine
Malcolm and Miller, (1972)	Cases 1-2: 16 tablets (800 mg), oral	Case series	N = 2 (M)	Mean age 21 yrs (SD=1)	Case 1: SUD (LSD, cannabis, mescaline, barbiturates, and cocaine); Case 2: SUD (cannabis, mescaline, LSD)	Cases 1-2: None	Cases 1-2: None	NA	Case 2: paranoia, visual hallucinations	Case 1: anxiety; visual and auditory hallucinations	NA	NA	NA
Rowe *et al*., (1997)	Cases 1-8: 15 tablets (750 mg), oral	Case series	N = 8 (F)	Age = 14-17 yrs	Cases 1-8: None	Cases 1-8: None	Cases 1-3: Cannabis; cases 4-8: none	NA	NA	Visual hallucinations	NA	Disorientation, confusion, slurred speech and abnormal behaviour	Symptomatic resolution with restututio ad integrum. No medical support needed
White *et al*., (2015)	Daily use of 20 or 30 tablets of DH (1,000-1,500 mg), oral	Case report	N = 1 (F)	Age = 38 yrs	Chronic schizophrenia, SUD (cocaine, methamphetamine, and cannabis)	Gastroesophageal reflux disease	None	NA	Persecutory delusion	Auditory, visual, and tactile hallucinations	NA	Irritability; disorientation and disorganized behaviour	Psychotic relapse with diagnosis of treatment-resistant psychosis
**Diphenhydramine (DPH)**													
Bonham and Birkmayer, (2009)	1.5 g/die, oral	Case Report	N = 1 (F)	Age = 34 yrs	Chronic undifferentiated schizophrenia and SUD (cocaine)	NA	None	NA	Concretism, ideas of reference, thought insertion, insight and judgment were impaired	Chronic auditory hallucinations.	NA	NA	Psychotic relapse
Phillips *et al*., (2014)	Oral	Case report	N = 1 (F)	Age = 13 yrs	Previous suicide attempts	NA	Bupropion, citalopram, acetaminophen, omeprazole, and naproxen	NA	NA	Unspecified hallucinations	NA	Severe psychomotor agitation	Brought to the ED following a polydrug overdose; several doses of lorazepam and physostigmine produced resolution of hallucinations and attenuation of the antimuscarinic symptoms. GABAergic agents were used later in the hospital course for presumed symptoms of serotonergic and adrenergic toxicity after resolution of antimuscarinic effects
Saran *et al*., (2016)	Oral	Case Report	N = 1 (M)	Age = 21 yrs	Previous drug-related admissions due to intoxications	None	None	NA	Unspecified psychosis	NA	NA	NA	Partial resolution with levetiracetam and phenytoin along with haloperidol and benzodiazepines for symptomatic management of ongoing psychosis. The IV administration of DPH resulted in immediate improvement. Then started on oral DPH, finally gradually reduced
**Promethazine**													
Abeysundera *et al*., (2021)	8 tablets of 25 mg over 4 days	Case Report	N = 1 (M)	Age = 48 yrs	Affective disorder type 2, social anxiety and alcohol use disorder	NA	Promethazine and alcohol	No	Persecutory delusions	Visual and tactile hallucinations	No	Promethazine-induced delirium	Patient gradually started to improve in mental state after 48 hours, which further supports the diagnosis of delirium. Insight improved and hallucinations slowly dissipated
Scott *et al*., (2007)	1,150 mg (46 tablets), oral	Case report	N = 1 (F)	Age = 14 yrs	Depression	Migraine	Cyproheptadine	NA	NA	Distressing visual hallucinations	NA	Delirium with unintelligible speech psychomotor agitation, confusion	Resolution of psychotic crisis in nine days after olanzapine
**Other**													
Alevizos, (2003)	D-norpseudo-ephedrine (35 mg/ml), 26.25 mg/day, Started from 26.25 mg/day and increased to 80 mg/day, oral	Case report	N = 1 (F)	Age = 45 yrs	None	None	None	Euphoria, insomnia, diminished sense of fatigue	Persecutory delusions, fear, disorganized behaviour; accelerated thinking	Auditory and visual hallucinations	NA	Withdrawal symptoms included: dysphoria, restlessness, impaired memory, bulimia, abnormal perceptions	Hospitalized and treated with haloperidol 20 mg/day and thioridazine 150 mg/day. Psychotic symptoms remitted rapidly, but after discharge from the hospital experienced depressive symptomatology with suicidal thoughts, which was remitted with clomipramine 100 mg/day. Many psychotic relapses concomitantly to the re-use of the drug. Failed to return to baseline functioning
Bachar *et al*., (2021)	Chlorpheniramine (CPA) and dextromethorphan (DXM); six tablets twice daily for the past 6 weeks	Case report	N = 1 (F)	Age: 31 yrs	Anxiety	None	Previous methamphetamine abuse	No	Unspecified acute psychosis, harmful ideation	Auditory hallucinations	No	Psychomotor agitation	After 24 hours of critical care support, a trial of sedation was attempted. A spontaneous awaken trial was performed. The patient awoke with normal mentation and appropriate behaviour
Das *et al*., (2017)	Chlorpheniramine; 60 mg/day, oral	Case Report	N = 1 (M)	Age = 34 yrs	Chlorpheniramine initiated as sleep aid, and then consumed at high dosage for 5 yrs	NA	None	Mood changes, euphoria, increased activity, anorexia, insomnia; feelings of blissfulness, increased religiosity	Delusions of grandeur	No	No	Abnormal behaviour, *e.g*., wandering around in night	Diagnosed as bipolar disorder induced mania
Ishigooka *et al*., (1991)	Methylephedrine, Codeine, Caffeine and Chlorpheniramine (BRON = oral	Survey	N = 44 (M: 32, F: 12)	Mean age: 25.3 yrs	None	NA	None	Group B depression, anxiety	Group A: Hallucinatory-paranoid state with delusional perception, persecutory delusions	Unspecified hallucinations	NA	Psychomotor excitement: irritability, and emotional disturbance	Accessed to mental institutions and rehabilitation centers for substance abuse
Leighton, (1982)	Triprolidine + Pseudoephedrine; (Actifed^®^) for many yrs (100-200 ml) at weekends. Increased intake to 200 ml a day, oral	Case report	N = 1 (M)	Age = 27 yrs	Bipolar disorder	None	None	NA	Paranoid symptoms, with ideas of influence	Auditory hallucinations	NA	NA	Presented at the psychiatric outpatient department, lithium treatment was continued as before and trifluoperazine 5 mg nightly added
Mohan *et al*., (2021)	Diphenhydramine and naproxen; surrounded by three empty bottles, each originally containing 80 caplets of naproxen sodium 220 mg and diphenhydramine hydrochloride 25 mg	Case Report	N = 1 (F)	Age = 22 yrs	Bipolar disorder	NA	Diphenhydramine and Naproxen	No	Altered mental status	Auditory hallucinations	No	No	Emergency providers should be familiar with diphenhydramine toxicity, especially the life-threatening neurologic consequences and risk of cardiovascular collapse
Pugh and Howie, (1986)	Triprolidine + Pseudoephedrine (Actifed^®^); 50-300 ml/day oral	Case report	N = 1 (F)	Age =21 yrs	Depression	None	None	NA	Unspecified psychotic symptoms	Auditory and visual hallucinations	NA	NA	NA; Treated with oral and depot phenothiazines in addition to supportive psychotherapy
Sullivan, (1996)	Pseudoephedrine; 60 mg, IV	Case report	N = 1 (M)	Age =18 yrs	Depression	None	None	NA	Paranoid delusions with fear	Visual and somatic hallucinations	NA	Psychomotor agitation	Complete recovery within 24 hours after treatment with IM Clopixol^®^ (50 mg)
Tennant, (1973)	DPH + Methaqualone (Mandrax^®^); unspecified dose, oral	Retrospective review (Jan-Jun 1972)	N = 67 (M). All patients were U.S. Army Soldiers in West Germany	Age range: 18 to 24 yrs	NA	None	Methaqualone, alcohol, cannabis	NA	Unspecified acute psychosis	NA	NA	Sedation, psychologic dependence, suicide attempt during intoxication (N=3), violence during intoxication (N=2)	Psychosis treatment consisted antipsychotic agents, such as chlorpromazine, IV fluids, and close observation; withdrawal symptoms required chlorpromazine or diazepam, and in some cases diphenylhydantoin
Tsay *et al*., (2014)	Codeine and DXM; oral	Retrospective review	N = 354 promethazine abuse and intentional misuse cases reported to the National Poison Data System (N = 95 promethazine alone; N = 259 promethazine in coformulation)	The sample considered 10 yrs and older subjects: exposures were prevalent among 10 to 19 yrs old and young adults (20s)	NA	NA	The most frequent co-formulates were codeine and DXM	NA	NA	Unspecified hallucinations	-	Agitation, confusion, slurred speech	Promethazine alone abuse was mostly managed in health care facilities, while promethazine in coformulation had more severe outcomes, requiring ED care management. Outcomes for both cases were up to moderate effects, and there were no reported deaths due to promethazine

**Abbreviations**: ADHD: attention deficit and hyperactivity disease; DH: Dimenhydrinate; DPH: ddiphenhydramine; DXM: dextromethorphan; ED: Emergency department; F: female; IV: intravenous; LSD: lysergic acid diethylamide ; M: male; NA: not applicable; NMDA: N-methyl-D-Aspartate; OTC: over-the-counter; PTSD: post-traumatic stress disorder; ROA: route of administration; SC: subcutaneous; SD: Standard Deviation; SUD: Substances Use Disorder.

**Table 2:** Summary of results related to psychotic outcome(s).

**Over the Counter Medication**	**Substance-induced Psychosis**	**Psychotic onset with Tendency to Chronicity**
*Dextromethorphan*	N = 16 (Akerman *et al*., 2010; Bernstein *et al*., 2019; Craig, 1992; Dilich and Girgis, 2017; Ghosh, 2011; Jamison and Vasudeva, 2009; Logan *et al*., 2012; Martinak *et al*., 2017; Miller, 2005; Modi *et al*., 2013; Roberge *et al*., 1999; Stanciu and Penders, 2015; Sharma *et al*., 2005; Walker and Yatham, 1993; Wolfe and Caravati, 1995; Ziaee *et al*., 2005)	N = 5 (Amaladoss and Brien, 2011; Butwicka *et al*., 2013; Hapangama and Kuruppuarachchi, 2011; Hinsberger *et al*., 1994; Polles and Griffith, 1996)
*Dimenhydrinate*	N = 1 (Rowe *et al*., 1997)	N = 0
*Diphenhydramine*	N = 2 (Phillips *et al*., 2014; Saran *et al*., 2016)	N = 0
*Promethazine*	N = 2 (Abeysundera *et al*., 2021; Scott *et al*., 2007)	N = 0

## LIST OF ABBREVIATIONS

ADHD: Attention Deficit And Hyperactivity Disease
DH: Dimenhydrinate
DPH: Ddiphenhydramine
DXM: Dextromethorphan
ED: Emergency Department
F: Female
IV: Intravenous
LSD: Lysergic Acid Diethylamide
M: Male
N/A: Not Applicable
NMDA: N-methyl-D-Aspartate
NR: Not Reported
OTC: Over-the-counter
PTSD: Post-traumatic Stress Disorder
ROA: Route of Administration
SC: Subcutaneous
SD: Standard Deviation
SUD: Substances Use Disorder

## SUPPLEMENTARY MATERIAL

PRISMA checklist is available as supplementary material on the publisher’s website along with the published article.
