# Supplementary material for: Over-the-counter Psychosis: A Systematic Review of the Misuse of Antihistamines, Cough Medicines, and Decongestants and the Risk of Developing Psychosis
Source: Curr Neuropharmacol. 2025 Feb 18;23(8):956–73. doi: 10.2174/011570159X344365250114064248 (PMC12174939; doi:10.2174/011570159X344365250114064248)
Supplement: Supplementary file 1 — PRISMA checklist is available as supplementary material on the publisher’s website along with the published article. [file CN-23-8-956_SD1.pdf]

Supplementary Material

Over-the-counter Psychosis: A Systematic Review of the Misuse of Antihistamines, Cough Medicines, and Decongestants and the Risk of Developing Psychosis

Alessio Mosca[1,*], Stefania Chiappini[2,3], Gianluca Mancusi[1], Andrea Miuli[1], Carlotta Marrangone[1], Rita Allegretti[1], Serena Panichella[1], Clara Cavallotto[1], John Martin Corkery[2], Mauro Pettorruso[1], Giovanni Martinotti[1] and Fabrizio Schifano[2]

[1]Department of Neuroscience, Imaging and Clinical Sciences, "G. D'Annunzio" University, 66100, Chieti, Italy; [2]Psychopharmacology, Drug Misuse and Novel Psychoactive Substances Research Unit, School of Life and Medical Sciences, University of Hertfordshire, AL10 9EU, Hertfordshire, UK; [3]UniCamillus International University of Medical Sciences, Via di S. Alessandro 8, Rome, Italy

1875-6190/25 $65.00+.00 **© 2025 Bentham Science Publishers**

PRISMA-DTA Checklist

Section/topic	#	PRISMA-DTA Checklist Item	Reported on page #
**TITLE / ABSTRACT**			1
Title	1	Over-the-counter psychosis: a systematic review of the misuse of antihistamines, cough Medicines, and decongestants and the risk of developing psychosis	1
Abstract	2	Abstract: Background: The widespread availability and accessibility of over-the-counter (OTC) medicines, play a vital role in modern healthcare systems, enabling individuals to manage minor health concerns independently. However, certain OTC medications possess pharmacological properties that render them susceptible to misuse and abuse, including stimulants, laxatives, sedatives, and opiate-containing products. Misuse involves improper dosage, duration, or indication, while abuse entails non-therapeutic use to achieve psychoactive effects or other illicit purposes, potentially leading to dependence and addiction.<br><br>This review explores the risk of developing psychotic symptoms associated with OTC drug misuse. Synthesizing existing literature, it comprehensively examines the relationship between antihistamines, cough medicines, and decongestants misuse, and the onset of psychotic symptoms.<br><br>Methods: A systematic literature review was carried out using Pubmed, Scopus and Web of Science databases through the following search strategy: (“diphenhydramine” OR “promethazine” OR “chlorpheniramine” OR “dimenhydrinate” OR “dextromethorphan” OR “pseudoephedrine” OR codeine- based cough medicines) AND (“abuse” OR “misuse” OR “craving” OR “addiction”) NOT review NOT (animal OR rat OR mouse). For data gathering purposes, the Preferred Reporting Items for Systematic Reviews and Meta-Analyses (PRISMA) was followed. Research methods were registered on PROSPERO (CRD42024527558).<br><br>Results: We analysed 46 relevant studies out of an initial pool of 2,677 articles. Key findings indicate that antihistamines, dextromethorphan, and other OTC drugs can induce psychotic symptoms, such as paranoia, hallucinations, and thought disorders when abused. Dextromethorphan is particularly associated with a chronic tendency towards psychosis, whereas other substances more commonly result in acute substance-induced psychosis.<br><br>Conclusion: The study underscores the necessity for increased awareness and specific interventions to address the misuse of OTC drugs and their potential to cause significant psychiatric disorders, emphasizing the broader public health implications of such misuse.	1
**INTRODUCTION**			2
Rationale	3	Over-the-counter drugs, widely consumed and readily available without the need for a prescription, often exert psychoactive effects on the central nervous system when taken at high doses and under specific conditions, such as in combination with alcohol or cannabis. Consequently, extensive research has focused on the misuse of drugs like Loperamide, laxatives, NSAIDs, and other analgesics. However, there remains limited understanding regarding the potential risk of developing psychosis as a result of misuse of these substances, or even precipitating the onset of schizophrenia. Therefore, the purpose of this review is to investigate the association between the misuse of over-the-counter drugs and the risk of psychosis development.	2
Objectives	4	The aim of this review is to investigate psychosis resulting from the misuse of over-the-counter drugs, specifically focusing on antihistamines (including diphenhydramine, promethazine, chlorpheniramine, and dimenhydrinate), cough medicines containing dextromethorphan, codeine-based cough medicines, and pseudoephedrine.	2
**METHODS**			2
Protocol and registration	5	Research methods were registered on PROSPERO (CRD42024527558).	3
Eligibility criteria	6	No restrictions were placed on the population of this review, we will include any original articles written in English (open-label or double-blind studies, prospective or retrospective observational studies, case series and case reports).	3
Information sources	7	The database selected for this review are: PubMed, Scopus and Web of Science	2
Search	8	("diphenhydramine" OR "promethazine" OR "chlorpheniramine" OR "dimenhydrinate" OR "dextromethorphan" OR "pseudoephedrine" OR Codeine-based cough medicines) AND ("abuse" OR	2

		"misuse" OR "craving" OR "addiction") NOT review NOT (animal OR rat OR mouse) NOT "in vitro"	
Study selection	9	Three independent reviewers will screen articles based on title and abstract removing:<br>1. Non-original research (e.g. review, commentary, editorial, book chapter)<br>2. No full-text article (e.g. meeting abstract)<br>3. Language other than English<br>4. Animal studies<br>5. OCT drugs are mentioned only as an example in the context of over-the-counter drugs misuse<br>6. Not deal with the misuse of OCT drugs<br>7. Not psychotic symptoms reported<br><br>First cross-check between reviewers<br><br>Selection phase: full-text screening of the eligible articles<br>1. Non-original research (e.g. review, commentary, editorial, book chapter)<br>2. No full-text article (e.g. meeting abstract)<br>3. OCT drugs are mentioned only as an example in the context of over-the-counter drug misuse<br>4. Not deal with the misuse of OCT drugs<br>5. Data not reported<br><br>6. Not psychotic symptoms reported<br><br>Final cross-check between reviewers	3
Data collection process	10	The search of results was carried out individually by three investigators (AM, AMo, and MCS) and supervised by SC and MP, doubtful cases will be discussed by the professors GM, MdG and FS. The selection and eligibility phase of the articles will be carried out independently by the three members selected and after subjected to a last cross-check.<br>We will include any data presented in original articles (open label or double-blind trials, prospective or retrospective observational studies, case series and case reports).	3
Definitions for data extraction	11	All original papers will be considered in this review (open-label or double-blind studies, prospective or retrospective observational studies, case series and case reports).	3
Risk of bias and applicability	12	The risk of bias will be measured independently by two members of the team (AM, AMo) supervised by SC and MP using the "Assessing risk of bias in included studies" edited by Julian PT Higgins and Douglas G Altman on behalf of<br>the Cochrane Statistical Methods Group and the Cochrane Bias Methods Group (Cochrane risk of bias tool)	3
Diagnostic accuracy measures	13	The data will be reported through a narrative summary	
Synthesis of results	14	The search of results was carried out individually by three investigators (AM, AMo, and TP) and supervised by SC and MP, doubtful cases will be discussed by the professors GM, and FS. The selection and eligibility phase of the articles will be carried out independently by the three members selected and after subjected to a last cross-check.Then, we will proceed to treat it descriptively as a narrative review. If applicable frequencies will be discussed as percentage, and the absolute number as mean ± standard deviation. All these procedures will being performed used SPSS 25.0.	3

Section/topic	#	PRISMA-DTA Checklist Item	Reported on page #
Meta-analysis	D2	None	
Additional analyses	16	None	
**RESULTS**			
Study selection	17	The search of results was carried out individually by three investigators (AM, AMo, and MCS).	3

Study characte-ristics	18	Original papers (open-label or double-blind studies, prospective or retrospective observational studies, case series and case reports).	3
Risk of bias and applicability	19	N/A	
Results of indi-vidual studies	20	Of the total 46 articles, 27 reported the use of dextromethorphan, 4 of dimenhydrinate, 3 of dip-henhydramine, 2 of promethazine, and 10 of other substances. Most studies were case reports and case series, except for 3 retrospective studies and one survey. Most subjects were male, while the age ranged from a minimum of 14, to a maximum of 69 years old. Regarding psychia-tric comorbidity, studies showed a wide variety, with substance use disorder being the most represented diagnosis (N=18), followed by mood disorders (N=12). Regarding medical comorbi-dities, most studies (N=36) did not report them. Regarding affective symptoms, most studies N=7 reported excitatory symptoms, and in N=8 cases, manic states. Regarding psychotic sym-ptoms, paranoia/paranoid delusion was the most reported thought disorder (N=18), while both visual and auditory hallucinations were found, either alone or in combination (N=32). Negative symptoms of schizophrenia were rarely reported, except in one case with apathy and anhedonia.	3
Synthesis of results	21	We analysed 46 relevant studies out of an initial pool of 2,677 articles.	3
Additional analysis	23	None	
DISCUSSION			5
Summary of evidence	24	Key findings indicate that antihistamines, dextromethorphan, and other OTC drugs can induce psychotic symptoms, such as paranoia, hallucinations, and thought disorders when abused. Dextromethorphan is particularly associated with a chronic tendency towards psychosis, whe-reas other substances more commonly result in acute substance-induced psychosis.	5
Limitations	25	The main difficulty regarding the literature on the misuse of OTC drugs concerns its heterogenei-ty, which is due to the lack of controlled clinical trials. In fact, most of the selected articles were case reports, case series, or observational studies.	5
Conclusions	26	The study underscores the necessity for increased awareness and specific interventions to ad-dress the misuse of OTC drugs and their potential to cause significant psychiatric disorders, emphasizing the broader public health implications of such misuse.	6
**FUNDING**			6
Funding	27	No founding	6

Adapted From: McInnes MDF, Moher D, Thombs BD, McGrath TA, Bossuyt PM, The PRISMA-DTA Group (2018). Preferred Reporting Items for a Systematic Review and Meta-analysis of Diagnostic Test Accuracy Studies: The PRISMA-DTA Statement. JAMA. 2018 Jan 23;319(4):388-396. doi: 10.1001/jama.2017.19163.

For more information, visit: **www.prisma-statement.org**.
